# Urgency overpowers cognitive control by amplifying cognitive processing asymmetries

**DOI:** 10.3758/s13414-025-03102-w

**Published:** 2025-07-13

**Authors:** Anika Krause, Christian H. Poth

**Affiliations:** 1 Differential Psychology, Personality Psychology and Psychological Assessment, Department of Psychology, Bielefeld University, Bielefeld, Germany; 2 Neuro-Cognitive Psychology, Department of Psychology, Bielefeld University, Bielefeld, Germany

**Keywords:** Cognitive control, Urgency, Flanker task, Processing asymmetries

## Abstract

Cognitive control is the functional backbone of intelligent behavior, because it allows to act according to one’s intentions, even when the environment elicits opposed behaviors. Recently, it has been shown that reactions under urgency in a cognitive control task are dominated by stimulus-driven information and goal-directed actions are overpowered, as reflected by a temporary drop in performance below chance level in conflict situations. This effect was shown for eye movements as well as manual cognitive control tasks. Crucially, most previous studies used tasks that involved a natural processing asymmetry between the stimulus-driven information and the goal-directed information, leaving it unclear whether urgency affects cognitive control in general. Here, we investigated whether urgency also impacts performance in tasks that evoke a stimulus-stimulus conflict between similarly processed stimuli. Therefore, urgency was applied to two Eriksen flanker tasks, one using color stimuli, the other one using letter stimuli. In both experiments, urgency did not lead to a drop in performance below chance level in conflict situations, meaning that goal-directed behavior could be maintained. In a third experiment, an Eriksen flanker task with letter stimuli and a stimulus-onset asynchrony (SOA) of 120 ms between the appearance of the flanker stimuli and the target stimulus, urgency evoked a large drop in performance below chance level. These results reveal that the effect of urgency on cognitive control is based on an amplification of cognitive processing asymmetries induced by urgency and is thus specific for tasks involving processing asymmetries, thereby evoking early-onset cognitive conflicts.

**Supplementary Information:**

The online version contains supplementary material available at 10.3758/s13414-025-03102-w.

## Introduction

Cognitive control ensures that in conflict situations humans can coordinate information from the environment with their internal goals, in order to react in a goal-driven manner (Cohen, 2017; Egner, 2017; Gratton et al., 2018; Miller & Cohen, 2001). Recently it has been shown that urgency opens up a time-window, in which the reaction in cognitive control tasks is mainly driven by stimulus information instead of goal-directed information (Krause & Poth, 2023; Poth, 2021; Salinas et al., 2019). Critically, previous studies used cognitive control tasks that involved the processing of spatial aspects of stimuli. Visual processing for spatial location has recently been shown to occur earlier and faster than processing for stimulus identity (Poth & Schneider, 2025). Therefore, the processing of spatial information in cognitive control tasks could have an advantage compared with other stimulus features. Thus, in terms of response selection, spatial information should have been processed with high efficiency and should have evoked a cognitive conflict early and fast (Pratte, 2021). This raises the question of whether the urgency-induced impairment can be transferred to tasks without the described processing asymmetry, which should evoke cognitive conflicts later in the processing chain. Indeed, we found no dominance of stimulus-driven over goal-directed processing in urgent cognitive control tasks without processing asymmetries, suggesting that the asymmetry was required for the effect. However, experimentally inducing an earlier processing of conflicting features made the dominance of stimulus-driven over goal-driven processing recur, suggesting that it was indeed the asymmetry in processing speeds for conflicting stimulus aspects that worked in concert with urgency to help stimulus-driven processing overcome cognitive control.

Since cognitive control allows us to execute goal-relevant actions even in the face of conflicting alternative actions, it can be seen as a crucial prerequisite for intelligent human behavior (Egner, 2017; Gratton et al., 2018). Cognitive control refers to a set of cognitive functions that serve to coordinate stimulus information from the environment (bottom-up information) with internal goals or tasks (top-down information) to enable a goal-directed action (Cohen, 2017; Egner & Hirsch, 2005). The relevance of cognitive control becomes particularly evident when bottom-up information and top-down information contradict and cause conflicting action tendencies (Botvinick et al., 2001; Henik & Tzelgov, 1982; Lu & Proctor, 1995; Navon, 1977; Simon, 1990; Stroop, 1935). The resulting cognitive conflict between these action tendencies can be resolved by cognitive control. Cognitive control allows us to suppress non-goal-directed actions in order to focus on and thus execute goal-directed actions (Cohen, 2017; Egner, 2008; Gratton et al., 2018).

**Fig. 1:**
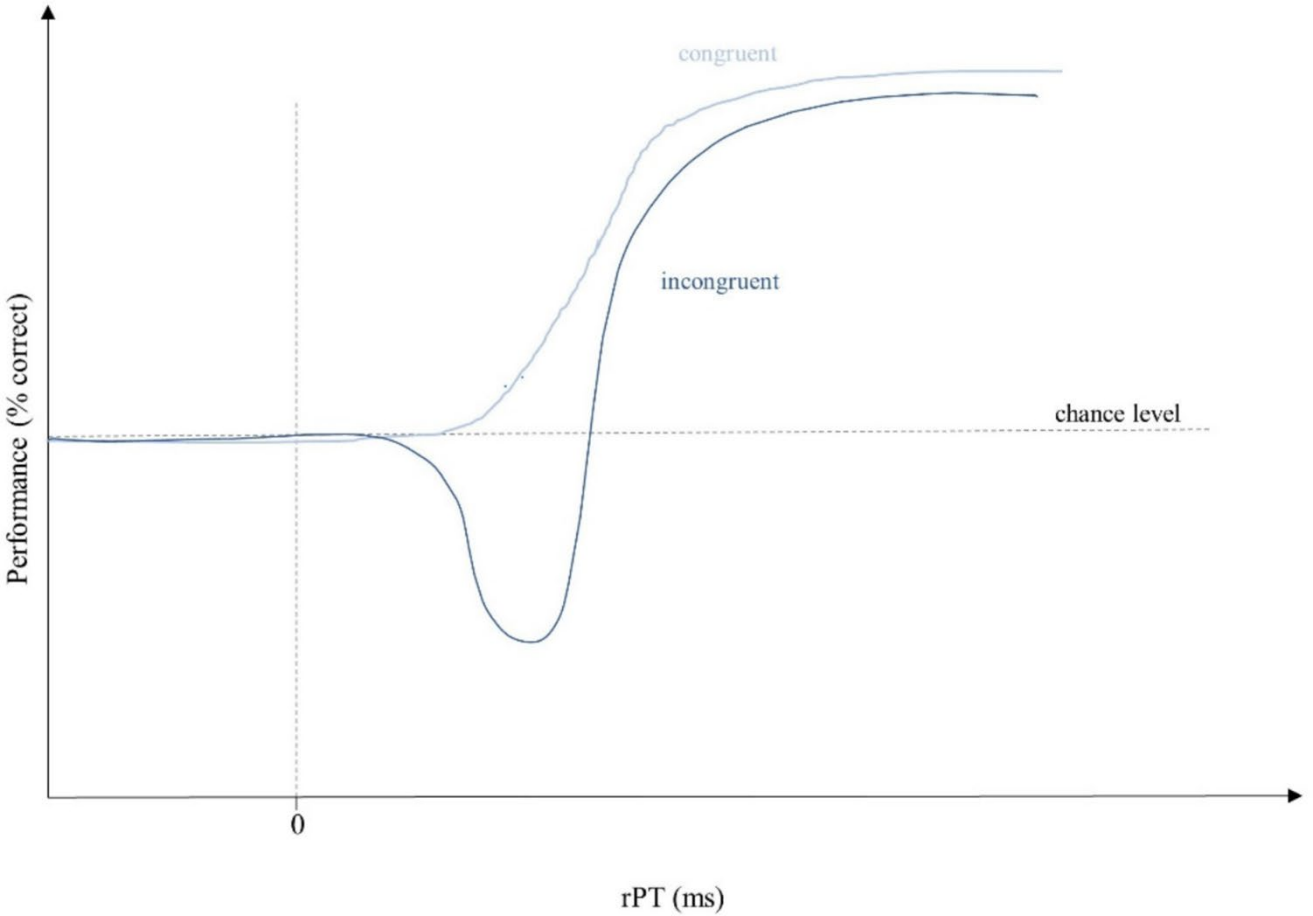
Tachometric functions of spatial cognitive control tasks (resketched after Poth, 2021). This figure illustrates what the tachometric functions would look like in a spatial cognitive control task like the Spatial Stroop task (cf. Krause & Poth, 2023; Poth, 2021). In both conditions performance fluctuates around chance for short raw processing times (rPTs). In the incongruent condition, performance then increases directly, approaching an asymptote close to near-perfect performance. Performance in the incongruent condition clearly drops below chance level for rPTs around 250 ms, before it also increases and approaches an asymptote. Mostly, performance in the incongruent condition tends to be marginally lower than performance in the congruent condition

Previous studies showed that cognitive control is heavily influenced by urgency (Krause & Poth, 2023; Poth, 2021; Salinas et al., 2019), akin to perceptual decision-making more generally (Shankar et al., 2011; Stanford et al., 2010; for a review, see Stanford & Salinas, 2021).Urgency can be defined as a type of time-pressure, induced by limiting the time available to process the target stimulus for responding (Poth, 2021; Salinas et al., 2019; Stanford & Salinas, 2021; Zhu et al., 2018). This was done by setting the response deadline to 1 s starting with a Go-Signal and simultaneously varying the duration of a gap between this Go-Signal and the onset of the target stimulus (Poth, 2021). Depending on the duration of the gap, the urgency was either low or high. Specifically, for long gap durations, there is only a small amount of time left in the response deadline to perceive the target, process it, and initiate the response, resulting in high urgency. However, urgency is low for trials with short gap durations, as there is a lot of time before the response deadline. Performance is then analyzed over the time of the raw processing time (rPT), which is defined as the time the target stimulus was shown within the time from Go-Signal to the response (Krause & Poth, 2023; Poth, 2021; Salinas et al., 2019). The results showed that the application of urgency on cognitive control tasks caused a dominance of stimulus-driven information over goal-directed information in a certain time interval, becoming visible in a drop in performance below chance level in conflict situations (Krause & Poth, 2023; Poth, 2021; Salinas et al., 2019). While performance in the congruent condition directly increased from chance level onwards, performance in the incongruent condition at first clearly dropped below chance level in a certain time window, before it recovered and then increased. This effect of urgency on cognitive control was first shown in an antisaccade task (Salinas et al., 2019). In this task, participants are instructed to perform a saccade away from a sudden stimulus onset (Hutton & Ettinger, 2006; Munoz & Everling, 2004; Salinas et al., 2019). However, under urgency, performance in the antisaccade task was shown to clearly drop below chance level (Salinas et al., 2019). Contrary to the instruction, participants mostly executed saccades towards the target stimulus in a certain time window (Salinas et al., 2019). With respect to manual tasks, this effect of urgency on cognitive control was shown for both spatial and non-spatial tasks (Krause & Poth, 2023; Poth, 2021) and also for tasks using different types of target stimuli (Krause & Poth, 2025). Here, too, urgency in conflict situations caused performance to drop below chance level for a certain time window, indicating that behavior was mostly stimulus-driven, although this contradicted the task goal. Figure 1 illustrates what the tachometric function looked like for spatial cognitive control tasks like the Spatial Stroop task.

Critically, the majority of previously used manual cognitive control tasks were spatial tasks, in which the spatial position of the target stimulus conflicts its meaning, such as the Spatial Stroop task or the Simon task (Krause & Poth, 2023, 2025; Poth, 2021). What these tasks have in common is that they evoke cognitive conflicts early and fast due to a natural processing asymmetry between the task-irrelevant stimulus information and the relevant information as it is reflected in the related delta plots (Pratte, 2021; Ridderinkhof, 2002). Delta plots illustrate the congruency effect over the reaction time (RT; De Jong et al., 1994). Tasks with conflict-driven stimulus information that is processed fast (such as the spatial position) tend to show negative-leaning curves in the delta plot and thus lead to a strong congruency effect in fast responses (Pratte et al., 2010; Ridderinkhof, 2002). This is because for short RTs the conflict-driven stimulus information has already been processed, while the slower top-down information has not yet been processed, so that the congruency effect is large since the conflict-driven stimulus information dominates the response. As the RT increases, the top-down information is processed, so that the congruency effect decreases. In the Numerical Stroop task used by Krause and Poth (2025), in which the conflict itself is not spatial but the response is determined by the spatial arrangement of the stimuli, it can also be assumed that the cognitive conflict peaks for fast RTs. The conflicting information was the physical size, which is also processed faster than the relevant numerical size, as shown by the fact that in a numerical Stroop task RTs for physical size comparisons are faster than RTs for numerical size comparisons (Dadon & Henik, 2017; Henik & Tzelgov, 1982).

Only Experiment 2 of Poth’s study used a truly non-spatial task, in which the spatial position of the stimulus does not drive the cognitive conflict, the Eriksen flanker task (Eriksen, 1995; Eriksen & Eriksen, 1974; Poth, 2021). In the Eriksen flanker task, a target stimulus is displayed in the center of the screen. The meaning of the target stimulus determines the correct response. However, the target stimulus is presented surrounded by flanker stimuli, which are shown in equal numbers to the right and left of the target stimulus (Eriksen, 1995; Eriksen & Eriksen, 1974). The meaning of the flanker stimuli may coincide with that of the target stimulus (congruent condition) or differ from it (incongruent condition). Thus, in the incongruent condition, the flanker stimuli elicit a different response to that of the target stimulus, resulting in a cognitive conflict between these response alternatives (Eriksen, 1995; Sanders & Lamers, 2002). This cognitive conflict is reflected in prolonged RTs as well as in an increased error rate observed in the incongruent condition, which is referred to as the *congruency effect* (Eriksen, 1995). In the classic Eriksen flanker task, the stimuli used were letters, but there are also variations of this task with other stimuli such as colors, shapes, arrows, faces, and even moving visual stimuli, amongst many more (Fenske & Eastwood, 2003; Hazeltine et al., 2000; Lange-Malecki & Treue, 2012; K. R. Ridderinkhof et al., 2021; Verbruggen et al., 2006). It can be assumed that the Eriksen flanker task does not involve any asymmetry in processing speed, since the tasks consist of a conflict between similarly processed stimuli.

However, Poth used an arrow version of the Eriksen flanker task (Poth, 2021; K. R. Ridderinkhof et al., 2021). Arrow stimuli have been shown to be highly associated with spatial directions, as they automatically favor responses that match their pointing direction, even if the arrow itself is task-irrelevant (Eimer, 1995; Hommel et al., 2001; Ristic & Kingstone, 2006). In the Eriksen flanker task, the precise characteristics of the stimulus material are highly relevant and have an influence on the evolution of the congruency effect that is observed (Pratte, 2021). Comparing the development of the congruency effect over time in Eriksen flanker tasks with different stimuli reveals differences in the delta plots. In an Eriksen flanker task with arrow stimuli the delta plot is an inverted U shape, whereas in an Eriksen flanker task with colors or letters positive-leaning delta plots occur (Pratte, 2021).

Thus, the delta plot in an Eriksen flanker task with arrows more closely resembles those of tasks in which spatial stimulus information elicits the conflict (Pratte, 2021; Pratte et al., 2010). With regard to the Eriksen flanker task, this indicates that in an Eriksen flanker task with arrow stimuli, the spatial associations of the arrow stimuli may induce early cognitive conflicts such as they occur in spatial cognitive control tasks like the Spatial Stroop task or the Simon task (Lu & Proctor, 1995; Pratte, 2021; Pratte et al., 2010; Simon, 1990). With higher-level conflicting information such as colors or letters, on the other hand, the greater congruency effects occur with later responses (Pratte, 2021), as has also been observed for other non-spatial cognitive control tasks, for example the color-word Stroop task (Pratte et al., 2010; Stroop, 1935).

Since most of the tasks in which the effect of urgency on cognitive control was observed were spatial cognitive control tasks or non-spatial cognitive control tasks with spatially associated stimuli such as arrows, it can be assumed that a (at least partly) negative delta plot occurs in all these tasks, meaning that the cognitive conflict is largest for short RTs. Thus, in all cognitive control tasks used so far, there seems to be a faster processing of the conflicting stimulus information, which favors early cognitive conflicts. This processing asymmetry between the conflicting stimulus information and the goal-driven stimulus information, which leads to the occurrence of an early cognitive conflict, could have conditioned the dominance of the stimulus-driven response over the task-driven response under urgency, leaving it unclear whether urgency also affects cognitive control for tasks without a processing asymmetry, with later cognitive conflicts arising.

Because of the limited time, in high-urgency situations primarily fast responses are required. If the cognitive conflict in fast responses is greatest in tasks which involve an asymmetry in processing speed between the conflicting stimulus information and the goal-directed stimulus information, the effect of urgency on cognitive control might be specific to these tasks. This study investigates whether urgency also evokes a dominance of the stimulus-driven reaction over the goal-driven reaction in a task that does not involve a processing asymmetry due to faster processing of the stimulus-driven information. In two experiments, the urgency paradigm used by Salinas et al. (2019) and Poth (2021) was applied to two Eriksen flanker tasks. Unlike Poth’s experiment, this study used an Eriksen flanker task with color stimuli (Experiment 1) and a classic Eriksen flanker task with letter stimuli (Experiment 2). Neither colors nor letters have as strong overlearned spatial associations as arrows. Thus, this study investigated whether the dominance of stimulus-driven information over goal-directed information evoked by urgency also occurs in tasks that do not involve a processing asymmetry between these information, so that the cognitive conflict arises later than in the tasks used previously.

However, while studies regarding delta plots can show how the classic flanker effect develops over the RT, the present study goes beyond this congruency effect. While in non-urgent flanker tasks the congruency effect is mainly represented by an increase in RT, the urgent flanker task does rely on errors instead of RT. Due to the limited time available, participants do not react slower, but instead make more errors in the incongruent condition. Importantly, urgency does lead to a drop in performance below chance level in the incongruent condition, indicating that participants mainly react as driven by the salient stimulus information but against their task goal. This qualitative effect would also be expected in the present study if urgency also impacts performance in tasks without a processing asymmetry. However, if there is no effect of urgency on cognitive control, we would still expect to observe a flanker effect. In this study, the flanker effect would be represented by a horizontal shift of the tachometric function. In the incongruent condition, performance would be delayed in comparison with the congruent condition.

For Experiment 1 and Experiment 2 it was assumed that for the congruent condition, in which no cognitive conflict is elicited, results from previous experiments will be replicated here. The tachometric function, showing the performance in dependence of the time available for processing the target and initiating the response, the rPT, should reveal a performance around chance level for negative to short rPTs, before performance increases and follows an asymptotic progression up to near-perfect performance. In the incongruent condition, however, where the target stimulus and the flanker stimuli evoke contradicting reactions, results could show two possible outcomes. If urgency also affects cognitive control for later arising cognitive conflicts, we would expect that in the incongruent condition performance should be around chance level first, and would then drop clearly below chance level before it recovers and increases asymptotically to near-perfect performance. In contrast, if the effect of urgency on cognitive control is specific for early-onset cognitive conflicts evoked due to processing asymmetries, the tachometric function should be similar to the one in the congruent condition. Performance should be around chance level first, before it approaches an asymptote close to near-perfect performance. This increase in performance, however, could be delayed in comparison to the congruent function. In non-urgent flanker tasks, RTs are usually shorter in the congruent condition than in the incongruent condition, which is referred to as the *flanker effect*. Here, the flanker effect could be reflected by a delayed increase in performance in the incongruent condition. The cognitive conflict has to be resolved first, before the correct response can be executed, which means that in the incongruent condition a longer rPT is required for the performance to increase.

Following up on Experiment 1 and 2, Experiment 3 specifically tested the idea that the dominance of stimulus-driven over goal-driven information was specific for early cognitive conflicts. Here, the processing asymmetry between the conflicting stimulus information and the goal-directed information was manipulated directly. Again, an Eriksen flanker task with letter stimuli was used, but in addition to the urgency paradigm a stimulus-onset asynchrony (SOA) variation (SOA of 0 or 120 ms) from the appearance of the flanker stimuli to the target stimulus was implemented. The aim was to artificially generate a processing asymmetry between the flankers and the target in the 120-ms SOA condition and thus create an early and large cognitive conflict.

Many previous studies have shown that the congruency effect in a classic Eriksen flanker task is larger for trials in which an SOA between the presentation of the flanker stimuli and the appearance of the target stimulus was introduced (Eriksen & Schultz, 1979; Flowers & Wilcox, 1982; Hübner & Töbel, 2019; Sidarus & Haggard, 2016). Thus, it was assumed that the implementation of a SOA should elicit a processing asymmetry between the flankers and the target, which should increase the cognitive conflict for fast reactions and thus evoke a dominance of the stimulus-driven information over the task-driven information for urgent trials.

In the congruent condition, performance should be around chance level for early rPTs. Then, with increasing rPT, performance should increase and approach an asymptote close to perfect performance. This should be equivalent for both the 0-ms SOA as well as the 120-ms SOA condition. In the incongruent condition, for a SOA of 0 ms, the results of Experiments 1 and 2 should be replicated. In the 120-ms SOA condition, however, performance should be around chance level at first, but should then drop to be clearly below chance level. After this drop, performance should recover, increase over chance level, and progress asymptotically.

## Methods

### Transparency and openness

All experiments were preregistered on the Open Science Framework (Experiment 1: https://osf.io/s4w3z; Experiment 2: https://osf.io/5qv2r; Experiment 3: https://osf.io/wydzq). The first experiment was pre-registered as part of a larger project, the further results of which will be published as part of another project. We report how we determined our sample size, all data exclusions (if any), all manipulations, and all measures in the study.

Data analysis was performed using R (4.1.0, https://www.R- project.org/; R Core Team, 2021). All data, analysis code, and research materials are available on the Open Science Framework (https://osf.io/bgt36/).

#### Participants

The experiments were performed by six participants each (Experiment 1: all female, aged 19–23 years, *M* = 20, *Md* = 19.5, *SD* = 1.55 years; Experiment 2: three female and three male, aged 20–31 years, *M* = 24.83, *Md* = 24, *SD* = 4.07 years; Experiment 3: four female and two male, aged 21–31 years, *M* = 25, *Md* = 24.5, *SD* = 4.24 years). The sample sizes for the experiments were chosen in advance and are based on the studies of Krause and Poth (2023), Poth (2021) and Salinas et al. (2019).

All three experiments were conceptualized as Small-N-Designs, in which a small number of participants completes a large number of trials (Anderson & Vingrys, 2001; Smith & Little, 2018). This approach is established in this research field and was also used in previous studies (Krause & Poth, 2023, 2025; Poth, 2021; Salinas et al., 2019). Each participant completed 5,940 trials (in Experiments 1 and 2) or 5,280 trials (Experiment 3). Thus, we collected 31,680 or 35,640 trials for each experiment. A Small-N-Design was chosen in order to use a parametric experimental approach. In order to assess the dependent variable as a psychometric function of the independent variable, a large number of interval-scaled levels of the independent variable is collected per participant. Following this approach, in this study the performance can be investigated over the time of the rPT using a tachometric function.

All participants had normal or corrected-to-normal vision as well as intact color vision. Written informed consent was given before participation. The experiments followed the ethical regulations of the German Psychological Society (DGPs) and were approved by Bielefeld University’s ethics committee.

#### Experimental setup

For all experiments, the participants were placed in a dimly lit room sitting in front of a computer screen at a distance of 57 cm (Experiment 1) or 71 cm (Experiments 2 and 3). Before starting the experiment, the computer screen was preheated in order to obtain a stable luminance value (Poth & Horstmann, 2017). In Experiment 1, a 47 cm × 29 cm monitor from Samsung Electronics Company (South Korea; model 2233RZ) with a refresh rate of 100 Hz and a resolution of 1,680 × 1,050 pixels was used. The screen was controlled by a graphics card of the type GK104 (NVIDIA, Santa Clara, CA, USA). In Experiments 2 and 3, the monitor was a CRT-Monitor from View Sonic (Brea, CA, USA; model Graphics Serie G90fB; 36 cm × 27 cm) with a refresh rate of 100 Hz (Experiment 2) or 85 Hz (Experiment 3) and a resolution of 1,024 × 768 pixels. The screen was controlled by a graphics card of the type GeForce GTX 970 (driver version 344.48, NVIDIA, Santa Clara, CA, USA). In Experiments 2 and 3, a chin-and-forehead-rest was used for stabilizing the participant’s head to ensure a standardized distance from the screen. Additionally, the eye movement of the participant’s right eye were recorded using an Eyetracker (EyeLink 1000, SR Research, Ontario, Canada) with a measuring rate of 1,000 Hz. Both Eyetrackers were set with a 9-point grid calibration. During data collection, for technical reasons the setup of Experiment 3 was changed to that of Experiment 2 without using the Eyetracker. For assembling the response, in all experiments a wired computer mouse was used.

The experiments were controlled by the Psychtoolbox3 (Brainard, 1997; Kleiner et al., 2007) and the Eyelink Toolbox (Experiment 2; Cornelissen et al., 2002) and were run using the program MATLAB versions R2019a (Experiment 1; The MathWorks, Natick, MA, USA) and R2014b (Experiments 2 and 3), respectively.

In all experiments, all stimuli were presented in front of a gray background (RGB = 128, 128, 128). The fixation stimulus was a black square (0.2° × 0.2° visual angle; RGB = 0, 0, 0; < 1 cd/m^2^), which was presented in the center of the screen. As feedback stimuli, either a smiling and sad looking smiley (Experiments 1 and 3) or a green star and a red exclamation mark (Experiment 2) were used.

The experiments consisted of an Eriksen flanker task (B. A. Eriksen & Eriksen, 1974). In an Eriksen flanker task the target stimulus is presented in the center of the screen. This stimulus was surrounded by flanker stimuli, which were presented in equal numbers to the right and left of the stimulus. These flankers were irrelevant to the task. Experiment 1 consisted of a color version of this task. Five colored squares (0.5° × 0.5° visual angle), of which the middle square represented the target stimulus, were displayed in the center of the screen. The squares could be either green (RGB = 0, 70, 0; 7.30 cd/m^2^) or red (RGB = 105, 0, 0; 7.35 cd/m^2^). The flanker stimuli always had the same color, which could match that of the target stimulus (congruent condition) or deviate from it (incongruent condition). The participants were instructed to press the right mouse button when the target stimulus was red and correspondingly the left mouse button when the target stimulus was green. The color of the flanker stimuli was irrelevant for the task. In Experiments 2 and 3, the classic version of the Eriksen flanker task with letter stimuli was applied. The stimuli were the letters S and H (written in Arial font, size 20 pt, RGB = 0, 0, 0; < 1 cd/m^2^). Five letters were presented; again the middle letter was the target stimulus. If the letter was an S, the left mouse button was to be pressed, and if it was an H, the right mouse button was to be pressed. The flankers could again be either congruent or incongruent with the target stimulus and were irrelevant for the task.

#### Procedure

In Experiments 1 and 2, participants completed five sessions each with 1,188 trials, resulting in a total of 5,940 trials per participant. One session was divided in nine blocks with 132 trials each with the opportunity of taking short breaks between these blocks. The first block of the first session (132 trials) was used as a practice block. Within the blocks, the possible combinations of all variables occurred equally often and were presented in randomized order.

**Fig. 2:**
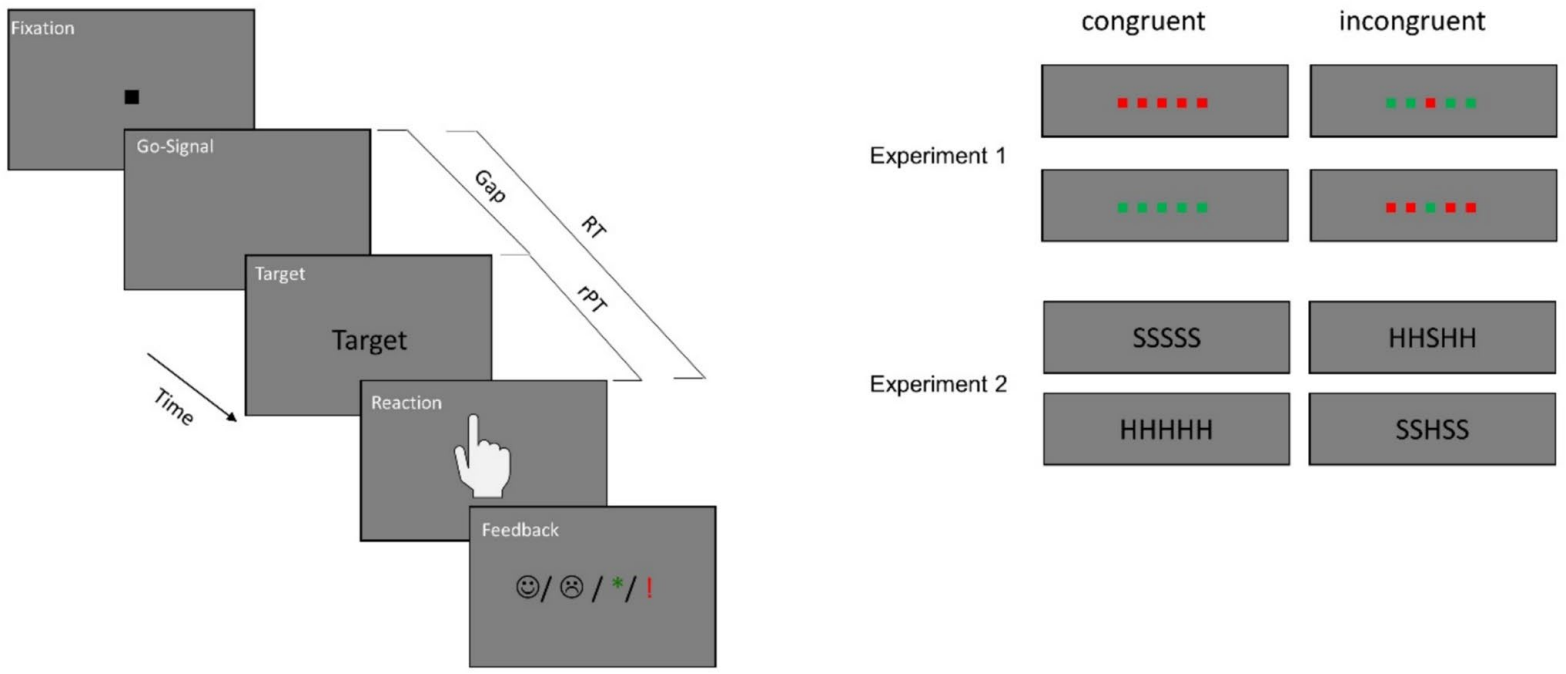
Experimental paradigms of Experiment 1 and Experiment 2. Each trial started with the presentation of the fixation stimulus for 350, 400, or 500 ms. Then the fixation stimulus disappeared, which represented the Go-Signal and marked the beginning of the response interval of 1,000 ms. After a variable gap of 0–950 ms (0, 100 200 … 900, 950 ms), the target appeared. In Experiment 1, the target was a red or green colored square presented in the middle of four flanker stimuli, which could match the targets color (congruent condition) or differ from it (incongruent condition). Reactions should match the color of the target, ignoring the flanker stimuli. In Experiment 2, a letter (S or H) served as target, and was also flanked by four matching (congruent condition) or deviating (incongruent condition) flanker letters. The reaction should correspond to the target letter, ignoring the flanker stimuli. After their reaction the stimuli disappeared and a feedback stimulus was shown for 750 ms, indicating the punctuality of the response. If participants reacted within the predetermined interval they received a positive feedback, which was either a smiling emoji (Experiments 1 & 3) or a green star (Experiment 2). If the response was delayed, they received a negative feedback – a sad emoji or a red exclamation mark

The experimental paradigm of Experiments 1 and 2 is shown in Fig. 2. Each trial started with the presentation of the fixation stimulus for 350, 400, or 500 ms. Afterwards, the fixation stimulus disappeared, which served as the Go-Signal and represented the beginning of the response interval. Participants were instructed to respond within 1,000 ms, beginning with the Go-Signal. Between the Go-Signal and the presentation of the target stimulus, a gap was implemented. The gap could last between 0 and 950 ms in 11 gradations (0, 100, 200, …, 900, 950 ms) and represented the degree of urgency. After the gap, the target stimulus and the flanker stimuli were presented in the center of the screen. The stimuli were shown until the response. After the reaction, the feedback stimulus was shown for 750 ms. The feedback stimulus indicated whether the reaction was executed within the 1,000 ms response interval. For the feedback, accuracy of the response was not considered. Urgency was manipulated by the duration of the gap. If the gap was short, a long time remained until the deadline for perceiving the stimuli, choosing the correct response, and executing it. However, if the gap was long, the time until the deadline was short, and the reaction had to be executed under urgency.

**Fig. 3:**
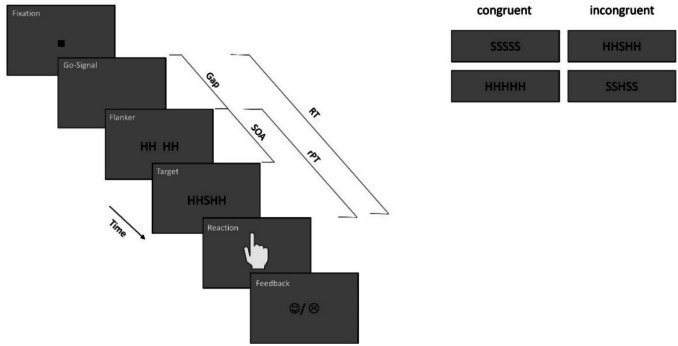
Experimental paradigm of Experiment 3. The trial procedure was similar to that of Experiment 2. In addition, a stimulus-onset asynchrony (SOA) was introduced between the appearance of the flanker stimuli and the appearance of the target stimulus, which could be either 0 ms or 120 ms. The figure shows a trial with an SOA of 120 ms

In Experiment 3 participants completed five sessions with 1,056 trials each, resulting in 5,280 trials per participant. Each session consisted of four blocks with 264 trials each. Again, participants had the opportunity to take short breaks between these blocks. The trials of the first block of the first session were considered as practice trials. Within the blocks, the possible combinations of all variables occurred equally often and were presented in randomized order. The experimental paradigm of Experiment 3 is presented in Fig. 3. The sequence of a trial in the 0 ms SOA condition was the same as in the previous experiments. In the 120 ms SOA condition, however, only the flanker stimuli were presented after the gap duration, while the target stimulus was presented with an SOA of 120 ms. The remainder of the procedure remained unchanged.

#### Data analysis

Data analysis was performed using R (4.1.0, https://www.R-project.org/; R Core Team, 2021). Data and analysis code are available on the Open Science Framework (https://osf.io/bgt36/). All experiments were analyzed following the same scheme. The practice trials were excluded before computing the analysis.

Initially, the rPT was calculated for each trial. The rPT was computed as the difference of the RT minus the gap duration (rPT = RT − gap duration). It represents the time available to perceive and process the target and to initiate the response. The rPT was considered as an independent variable in the following analyses (Salinas et al., 2019). Performance in terms of accuracy was calculated as the proportion of correct reactions. For the further analyses, only trials between −200 and 1,000 ms rPT were considered. Negative rPT values refer to responses that were executed before target presentation. As such, these reactions are guesses rather than stimulus-driven responses. We excluded trials with rPTs longer than 1,000 ms since they represent reactions outside the response deadline even for trials with a gap duration of 0 ms. For all experiments, tachometric functions, illustrating the proportion of correct responses in dependence of the rPT were plotted. The functions were plotted separately for the congruent and the incongruent conditions. Since the tachometric analysis requires a large number of trials, the analysis was performed on the aggregated data. Importantly, this was only done after ensuring that all participants showed the qualitatively same course of the performance over the rPT (Farrell & Lewandowsky, 2018). For rPTs from −200 to 1,000 ms, the average proportion of correct answers was calculated for a running bin of 1 ms. Afterwards, using the loess-function of R (span-parameter 0.2), the performance was locally regressed on these rPT bins.

To compare the minima of the tachometric functions between the congruency conditions, a permutation test was calculated, which is a robust non-parametric statistical method. The permutation test locates the original difference of the minima between the congruent and the incongruent condition in a distribution of effects from a 1,000-fold reanalysis of the raw data with a randomized labelling of the conditions. Since the global minima of the curves could be in a negative range of the rPT, only values with an rPT larger than 0 ms were included for the analysis of the minima. Negative rPT values represent responses that were executed before the target appeared and thus cannot be considered as being stimulus-driven. Furthermore, in order to test if performance in the incongruent condition does not only differ from the congruent condition but also drops below chance level, the minima of the congruent and the incongruent condition were tested against chance level (0.5) using a permutation test. Then, for visualizing if there is a time-window in which the tachometric function drops meaningfully below chance, we additionally computed 95% binomial confidence limits around chance using the formula CI = 1.96 * ((p*(1-p)/n) ^ (1/2)), where p is defined as the probability for a correct reaction (here chance level) and n as the number of trials on which that probability is based (cf. Macmillan & Creelman, 2005). The confidence limits were then plotted as a confidence band around chance. Additionally, a Bayesian analysis was computed. The performance in terms of the proportion of correct responses was calculated for 100-ms time-bins of the rPT. Using the proportionBF function of the BayesFactor package (Morey & Rouder, 2022), a Bayes factor was calculated for each time-bin which assessed the statistical evidence for the hypotheses that (a) performance was below versus identical to the chance level of 0.5, and (b) above versus identical to chance level of 0.5. In the figures, Bayes factors have been logarithmized decadally for better presentation. Bayes factors of 0 and infinity have been retained.

In Experiment 3, all these analyses were performed separately for the 0-ms SOA condition and the 120-ms SOA condition. Since the rPT should represent the time interval from the presentation of the target stimulus to the reaction, it was calculated as rPT = RT – gap −120 in the 120-ms SOA condition in order to consider the SOA between the flanker stimuli and the target stimulus. In addition, the minima of the incongruent functions of the two SOA conditions were compared by calculating a permutation test.

## Results

### Experiment 1

**Fig. 4:**
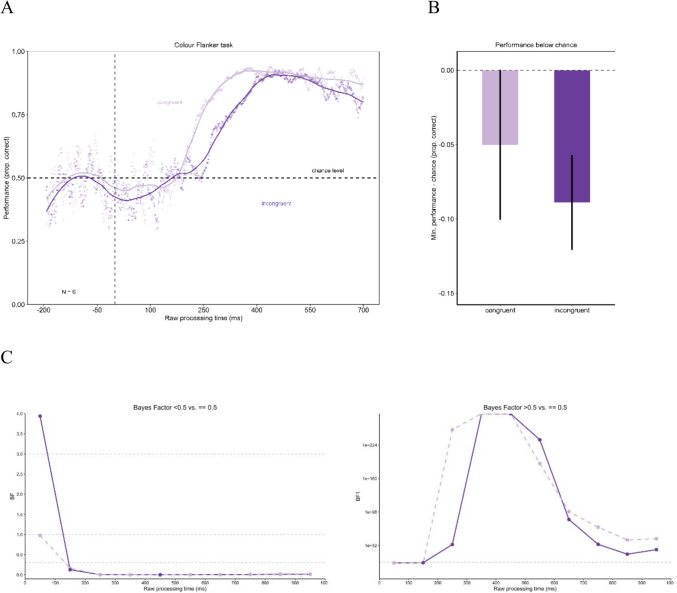
Urgency does not evoke dominance of stimulus-driven information in Experiment 1. In **Panel A** the tachometric functions for the congruent and the incongruent condition of the Eriksen flanker task with color stimuli are shown. In both conditions, performance starts around chance level and then rises with increasing raw processing time (rPT). Performance in the congruent condition rises earlier than in the incongruent condition. For long rPTs, the performance declines again. **Panel B** shows the minima of the tachometric functions for the congruent and the incongruent condition. The incongruent condition evoked no significantly larger drop below chance level. Error bars are standard errors from a bootstrap. **Panel C** displays the Bayes factors for 100-ms time-bins of the rPT

The results of Experiment 1 are shown in Fig. 4. Panel A shows the tachometric functions representing the progression of performance over the rPT. The tachometric functions in Experiment 1 show a similar pattern for the congruent and the incongruent conditions. Both functions initially fluctuate unsystematically around chance level for negative and short rPTs. From about 200-ms rPT, the tachometric function of the congruent condition rises above chance level and then proceeds monotonically increasing to a high performance. For particularly long rPTs, the performance decreases slightly. In the incongruent condition, the performance remains close to chance level for a slightly longer period before rising above chance level approximately 50 ms later. Here, too, the curve exhibits a monotonic increase, reaching a high point of performance before performance slight declines. The performance in the incongruent condition remains lower than in the congruent condition. The difference between the minima of the congruent condition (minimum = 0.44) and the incongruent condition (minimum = 0.41) was compared using a permutation test for reasons of investigating a significantly deeper drop in performance in the incongruent condition. The minima are shown in Fig. 4B. The permutation test revealed no significant difference between the minima with *p* = 0.208. When comparing the minimum of the incongruent condition against chance level (0.5), the permutation test did not reveal a significant difference (*p* = 0.481). Furthermore, the minimum in the congruent condition was not significantly different from chance level (permutation test with *p* = 0.139). The Bayes factors assessing evidence for performance being below/above chance versus identical to chance for time-bins of the rPT are presented in Fig. 4C. The Bayes factor confirms the reported results.

**Fig. 5:**
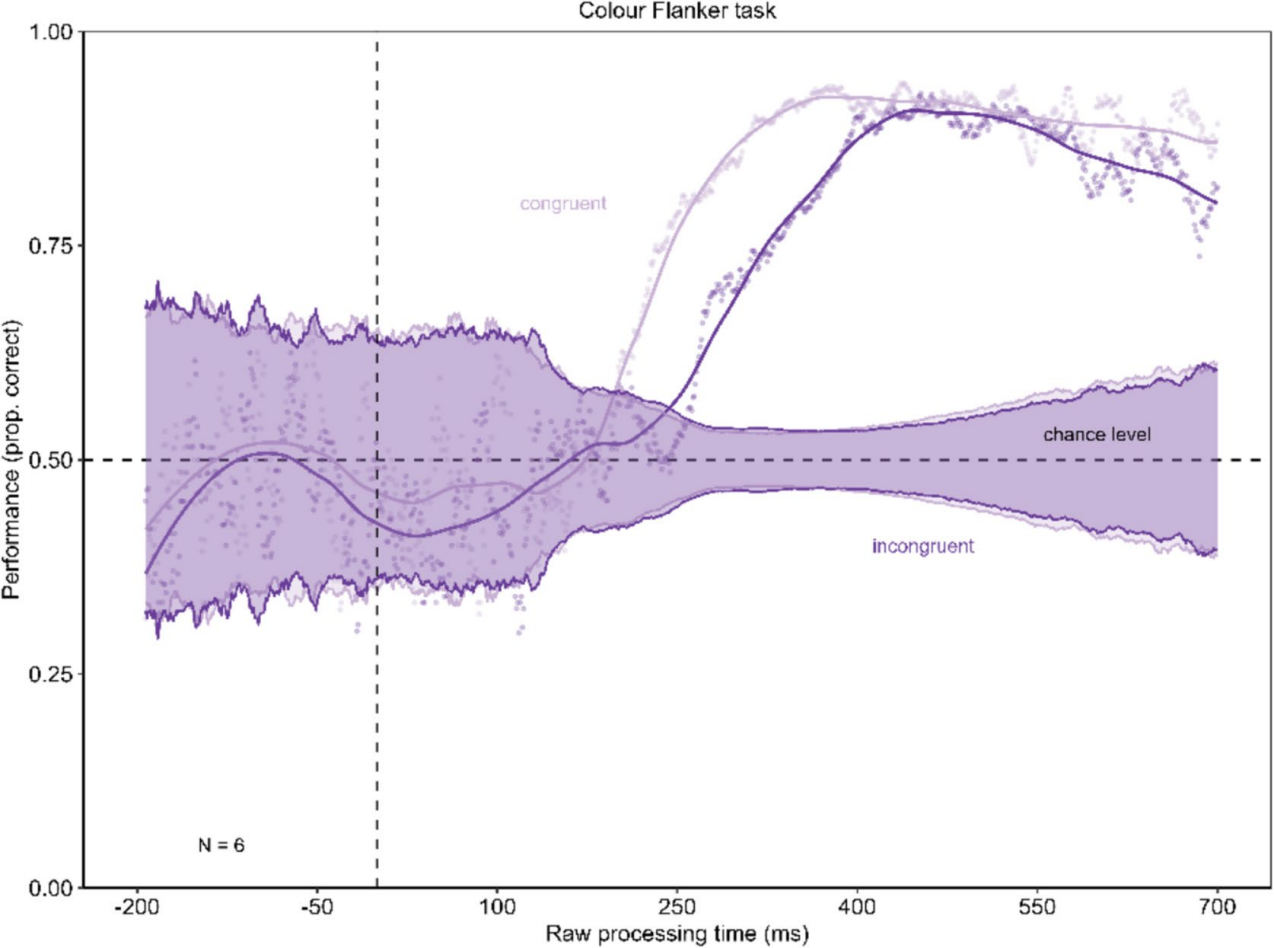
Confidence band around chance level for tachometric functions in Experiment 1. This figure presents the tachometric functions with a confidence band around chance level. The confidence limits were calculated using the formula CI = 1.96 * ((p*(1-p)/n) ^ (1/2)), where p is defined as 0.5 (chance level) and n as the number of trials on which that probability for a correct reaction is based (Macmillan & Creelman, 2005). The tachometric functions remain within this confidence band until performance increases, meaning it does not drop below chance level

When a 95% binomial confidence band is plotted around chance level, the tachometric function remains within this confidence band until it increases from approximately 250 ms onwards (see Fig. 5). This means that performance is not significantly below chance before it increases.

In the Eriksen flanker task with color stimuli, urgency does not seem to lead to a drop in performance under chancel level in the incongruent condition. Urgency does not seem to open up a time window where the reaction is mainly stimulus-driven. Even under urgency, goal-directed action can be maintained.

## Experiment 2

**Fig. 6:**
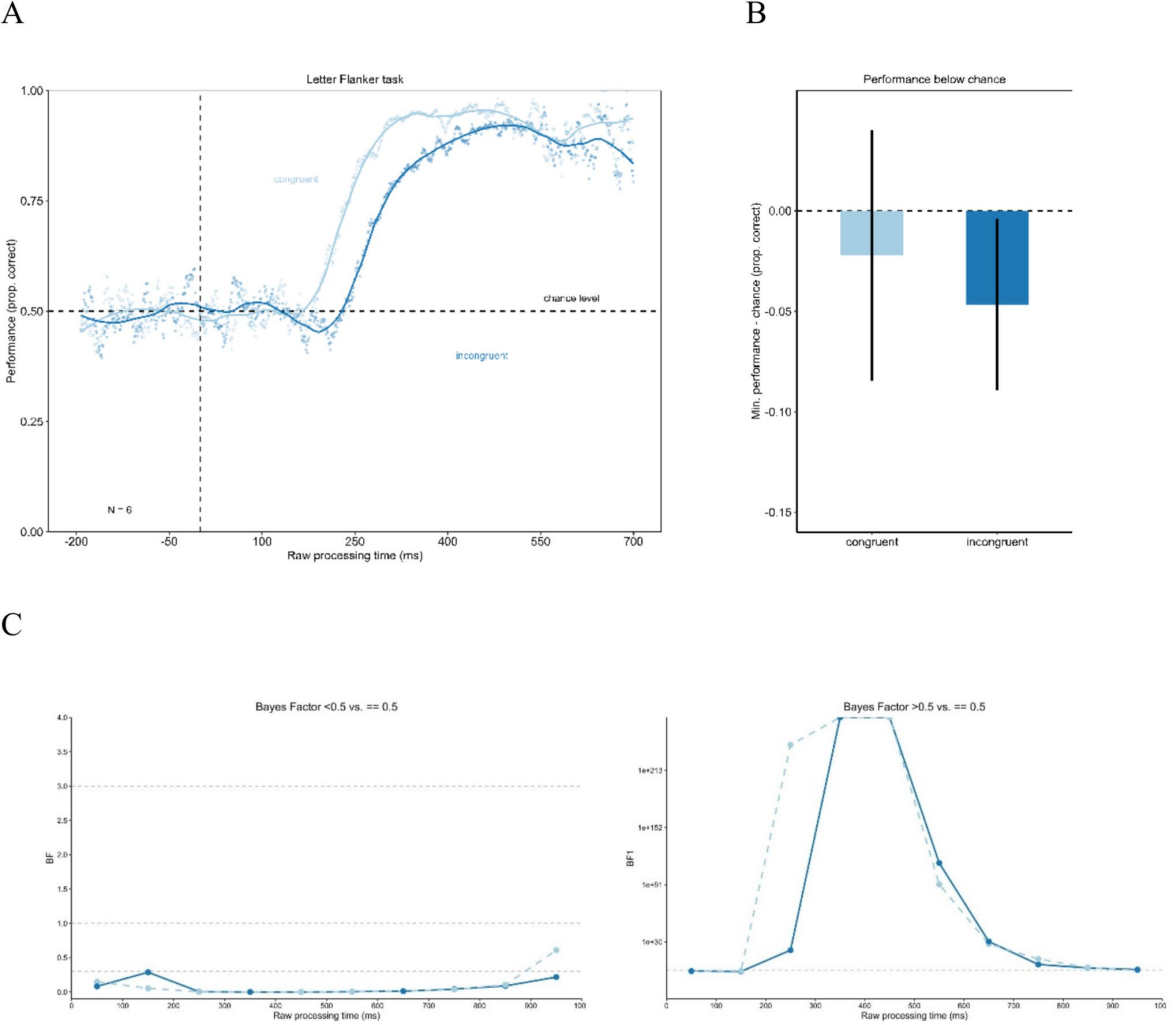
Urgency does not evoke dominance of stimulus-driven information in Experiment 2. **Panel A** shows the tachometric functions for the congruent and the incongruent condition of the Eriksen flanker task with letter stimuli. In both conditions, performance starts around chance level and then rises with increasing raw processing time (rPT). Performance in the incongruent condition remains around chance level longer before it increases. For long rPTs, the performance declines again. **Panel B** shows the minima of the tachometric functions for the congruent and the incongruent condition. The drop in performance below chance level was not significantly larger in the incongruent condition. Error bars are standard errors from a bootstrap. **Panel C** displays the Bayes factors for 100-ms time-bins of the rPT

The results of Experiment 2 are shown in Fig. 6. The tachometric functions (see Fig. 6A) show the same pattern as in Experiment 1. Initially, both the congruent condition function and the incongruent condition function fluctuate around chance level. From approximately 200 ms rPT onwards the curve corresponding to the congruent condition ascends, while that of the incongruent condition remains at chance level for an additional 50 ms before also ascending. Both curves attain a high performance level; however, the performance of the incongruent condition consistently remains lower than that of the congruent condition. With later rPTs, both curves decrease slightly. To test for a significant difference in the drop in performance below chance level, the minima of the congruenct conditions were compared using a permutation test (minimum congruent condition = 0.48; minimum incongruent condition = 0.45; see Fig. 6B). The permutation test showed no significant difference between those minima with *p* = 0.395. Additionally, the minimum in performance in the congruent (permutation test with *p* = 0.078) as well as in the incongruent condition (permutation test with *p* = 0.126) did not differ significantly from chance. The Bayes factors assessing evidence for performance being below/above chance versus identical to chance for time-bins of the rPT are presented in Fig. 6C. The Bayes factor confirms the reported results.

**Fig. 7:**
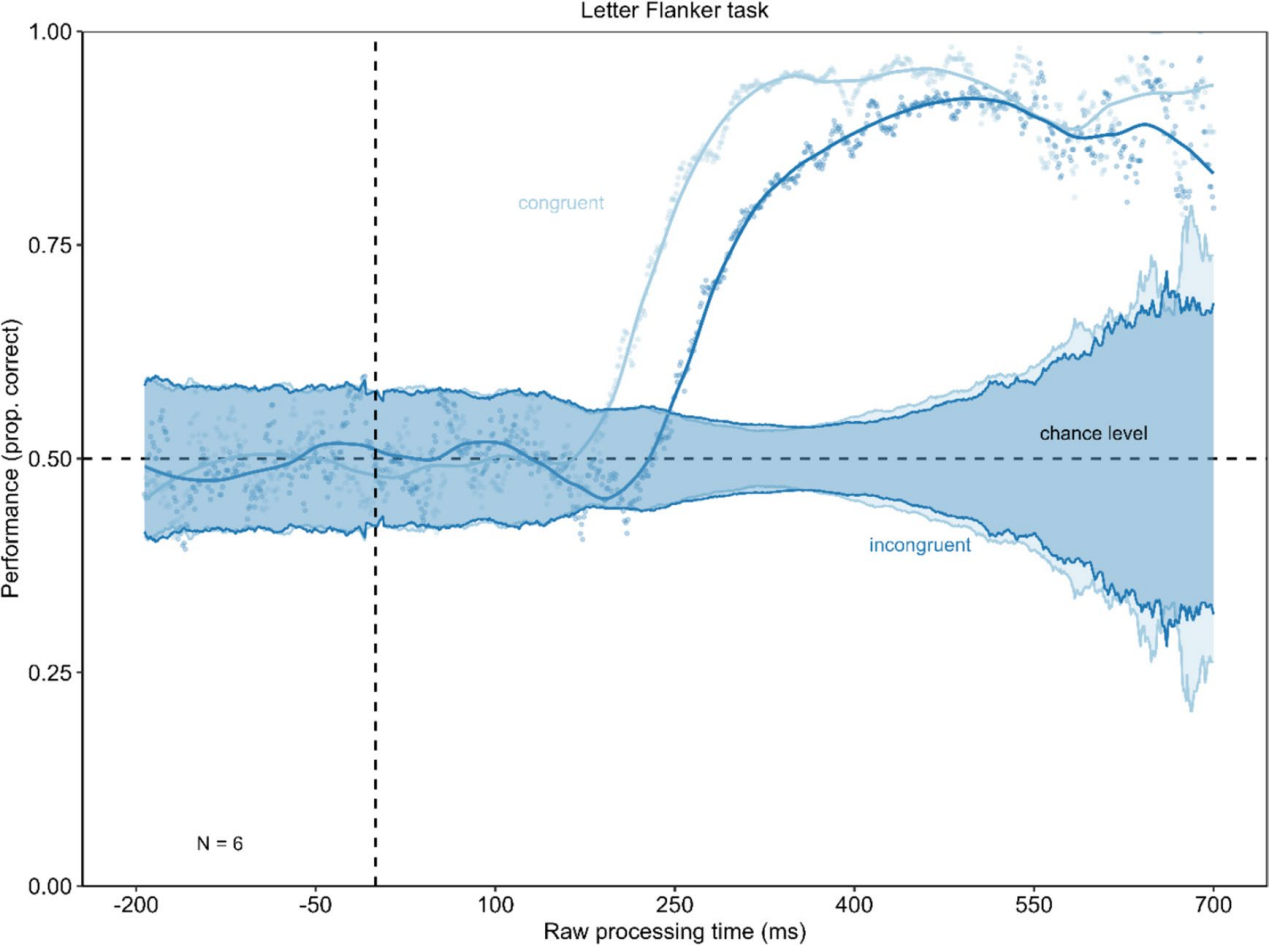
Confidence band around chance level for tachometric functions in Experiment 2. This figure presents the tachometric functions with a confidence band around chance level. The confidence limits were calculated using the formula CI = 1.96 * ((p*(1-p)/n) ^ (1/2)), where p is defined as 0.5 (chance level) and n as the number of trials on which that probability for a correct reaction is based (Macmillan & Creelman, 2005). The tachometric functions remain within this confidence band until performance increases, meaning it does not drop below chance level

When a 95% binomial confidence band is plotted around chance level, the tachometric function operates within these confidence limits, before performance increases up to near-perfect performance (see Fig. 7). Performance does not exhibit a drop below chance. level This finding is consistent with the results reported previously.

Experiment 2, an Eriksen flanker task with letter stimuli, also showed no drop in performance below chance level for the incongruent condition in high-urgency situations, replicating results from Experiment 1. In the Eriksen flanker task, urgency does not seem to impact cognitive control. Reactions are not dominated by the stimulus information only and are still goal-directed.

## Experiment 3

The 0-ms SOA condition of Experiment 3 replicated the results of Experiments 1 and 2. In the congruent condition, performance was around chance for short rPTs. With increasing rPT, performance also increased up to near-perfect performance. In the incongruent condition, performance was likewise around chance for the short rPTs. Performance increased a little later than in the congruent condition, but progressed equally until it reached near-perfect performance. A permutation test was calculated to test for a difference in the drop below chance level between the congruent and the incongruent condition. The minimum of the congruent condition was significantly deeper than in the incongruent condition (minimum congruent condition = 0.4; minimum incongruent condition = 0.49), with p < 0.001. The permutation test comparing the minimal performance in the conditions against chance level, revealed that performance does not drop below chance in the 0-ms SOA condition for both congruent (*p* = 0.239) and incongruent (*p* = 0.086) trials.

**Fig. 8:**
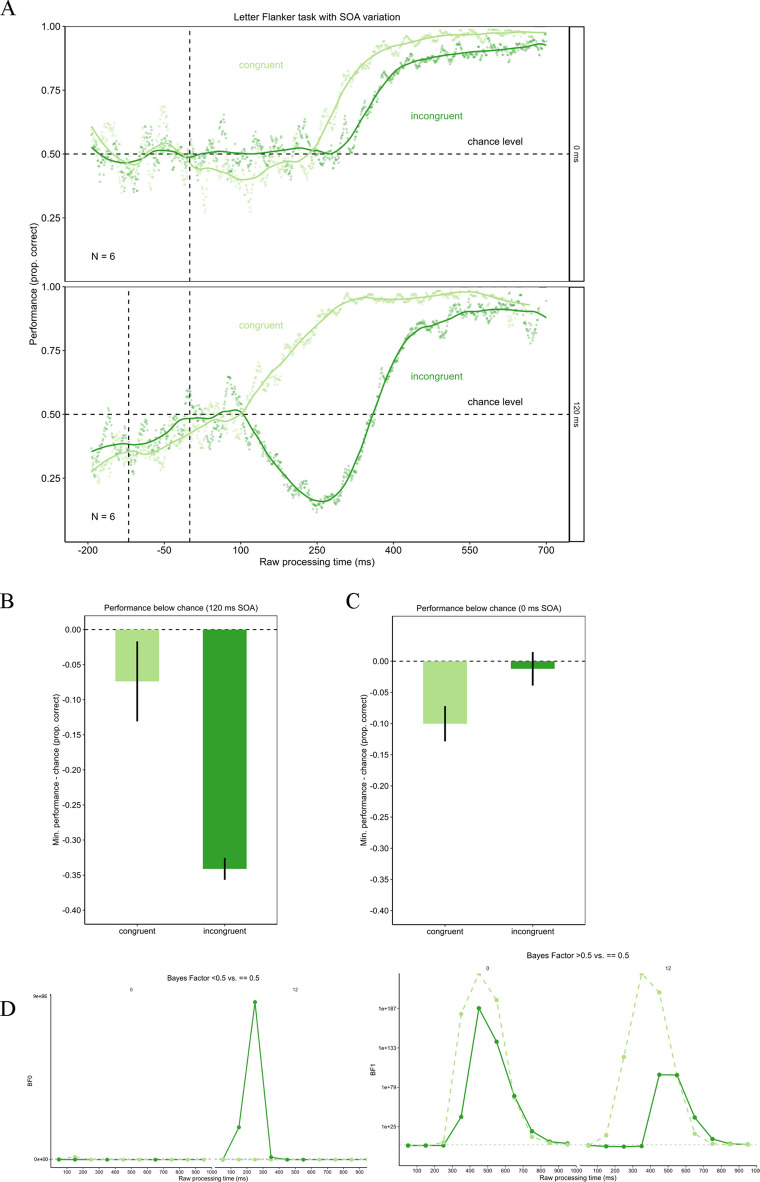
Urgency evokes dominance of stimulus-driven information in the 120-ms stimulus-onset asynchrony (SOA) condition. **Panel A** shows the tachometric functions for the congruent and the incongruent condition of the Eriksen flanker task with letter stimuli for the 0-ms SOA condition as well as the 120-ms SOA condition. In the 120-ms SOA condition, the first dashed line marks the onset of the flanker stimuli and the second dashed line marks the onset of the target stimulus. For both SOAs, performance in the congruent condition starts around chance level and then rises with increasing raw processing time (rPT). In the incongruent condition, performance differs between the SOA conditions. For the 0-ms SOA, performance starts around chance level and directly increases. In the incongruent condition with a 120-ms SOA, performance drops clearly below chance level, before it increases. **Panels B** and **C** show the minima of the tachometric functions for the congruent and the incongruent condition for the 0-ms SOA condition (**Panel B**) and the 120-ms SOA condition (**Panel C**). For a SOA of 0 ms, performance is further below chance in the congruent condition. In the 120-ms SOA condition, performance in the incongruent condition drops further below chance. Error bars are standard errors from a bootstrap. **Panel D** displays the Bayes factors for 100-ms time-bins of the rPT

For trials with a SOA of 120 ms the results differed. The congruent condition progressed just as in the 0-ms SOA condition and the previous experiments. In the incongruent condition, however, performance also fluctuated around chance level for short rPTs. For a time window around 400 ms, rPT performance dropped clearly below chance. After this drop, the performance recovered and increased. Comparing the depth of the drop below chance level, the performance dropped deeper in the incongruent condition (minimum = 0.16) than in the congruent condition (minimum = 0.43). This difference was statistically significant, *p* < 0.001. These results can be seen in Fig. 8. Also, in the congruent condition for the 120-ms SOA performance was not different from chance (permutation test with *p* = 0.285). However, in the incongruent 120-ms SOA condition, performance dropped significantly below chance (*p* < 0.001). The Bayes factors assessing evidence for performance being below/above chance versus at chance for time-bins of the rPT are presented in Fig. 8D. The Bayes factor confirms the reported results.

These results again replicate that urgency does not prevent goal-directed actions in the Flanker task when stimuli are processed similarly. With regard to the lower minimum in the congruent condition than in the incongruent condition for the 0-ms SOA, we conducted additional analyses which indicate an effect of handedness. It seems that in trials with high urgency, where participants were forced to initiate their response before the target was presented, they did this not by random guessing but instead preferred a response with their dominant hand. This remarkable feature is examined in more detail in the Online Supplemental Material.

**Fig. 9:**
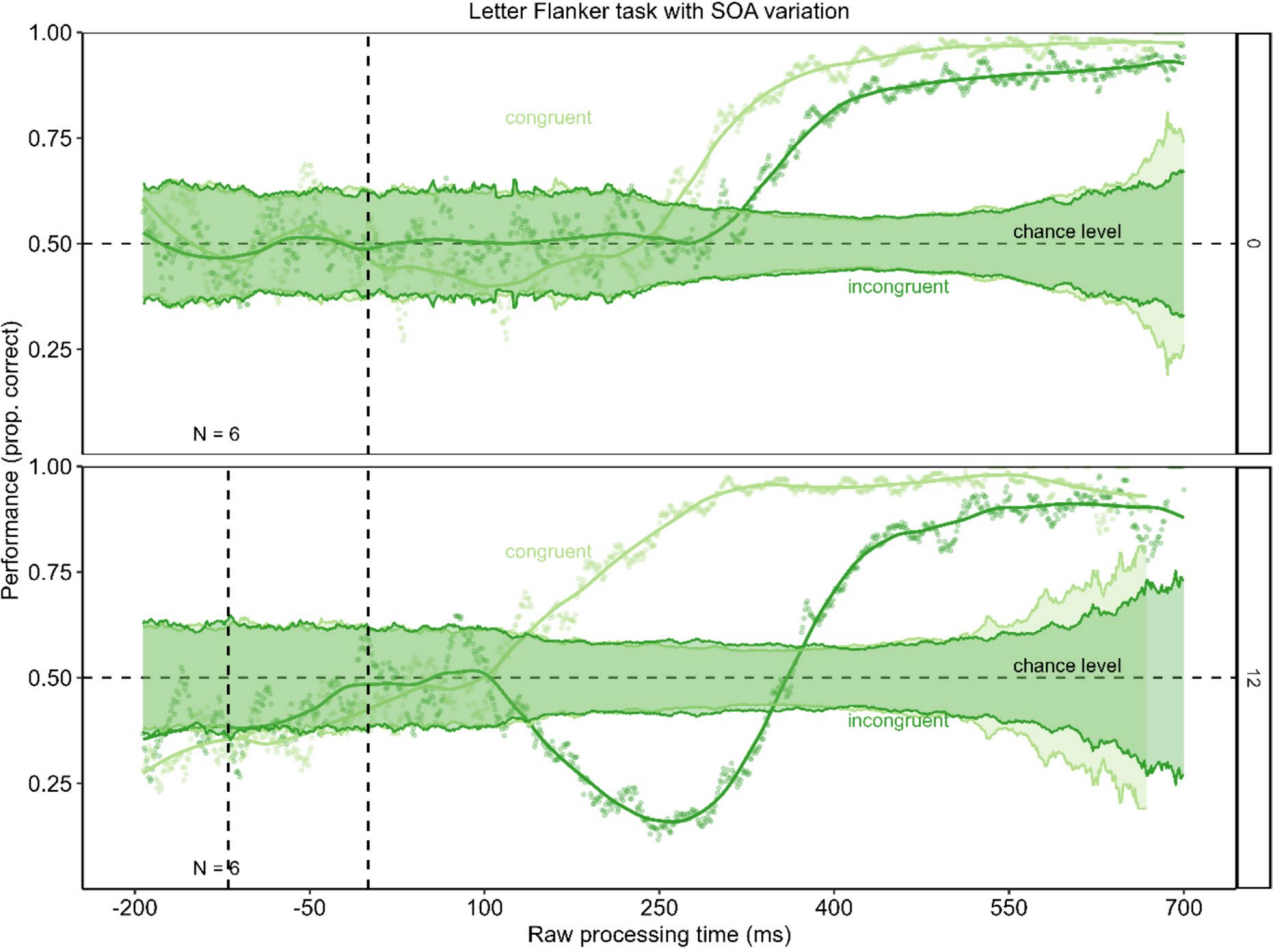
Confidence band around chance level for tachometric functions in Experiment 3. This figure presents the tachometric functions with a confidence band around chance level. The confidence limits were calculated using the formula CI = 1.96 * ((p*(1-p)/n) ^ (1/2)), where p is defined as 0.5 (chance level) and n as the number of trials on which that probability for a correct reaction is based (Macmillan & Creelman, 2005). For the 0-ms stimulus-onset asynchrony (SOA) condition the tachometric functions remain within this confidence band until performance increases, meaning it does not drop below chance level. Similar results can be found for the congruent 120-ms SOA condition. However, in the incongruent 120-ms SOA condition, performance does drop significantly below chance

This result is supported by additional analyses. When plotting the 95% binomial confidence limits around chance (see Fig. 9), the tachometric functions for the 0-ms SOA condition (both congruent and incongruent) only exceed the confidence band when performance increases from an rPT of approximately 250 ms onwards. In the 120-ms SOA condition, the congruent condition exhibited a similar pattern; however, in the incongruent condition, a drop below the chance level was observed, which was clearly outside the confidence interval. Consequently, this decline can be interpreted as being significantly different from chance level.

**Fig. 10:**
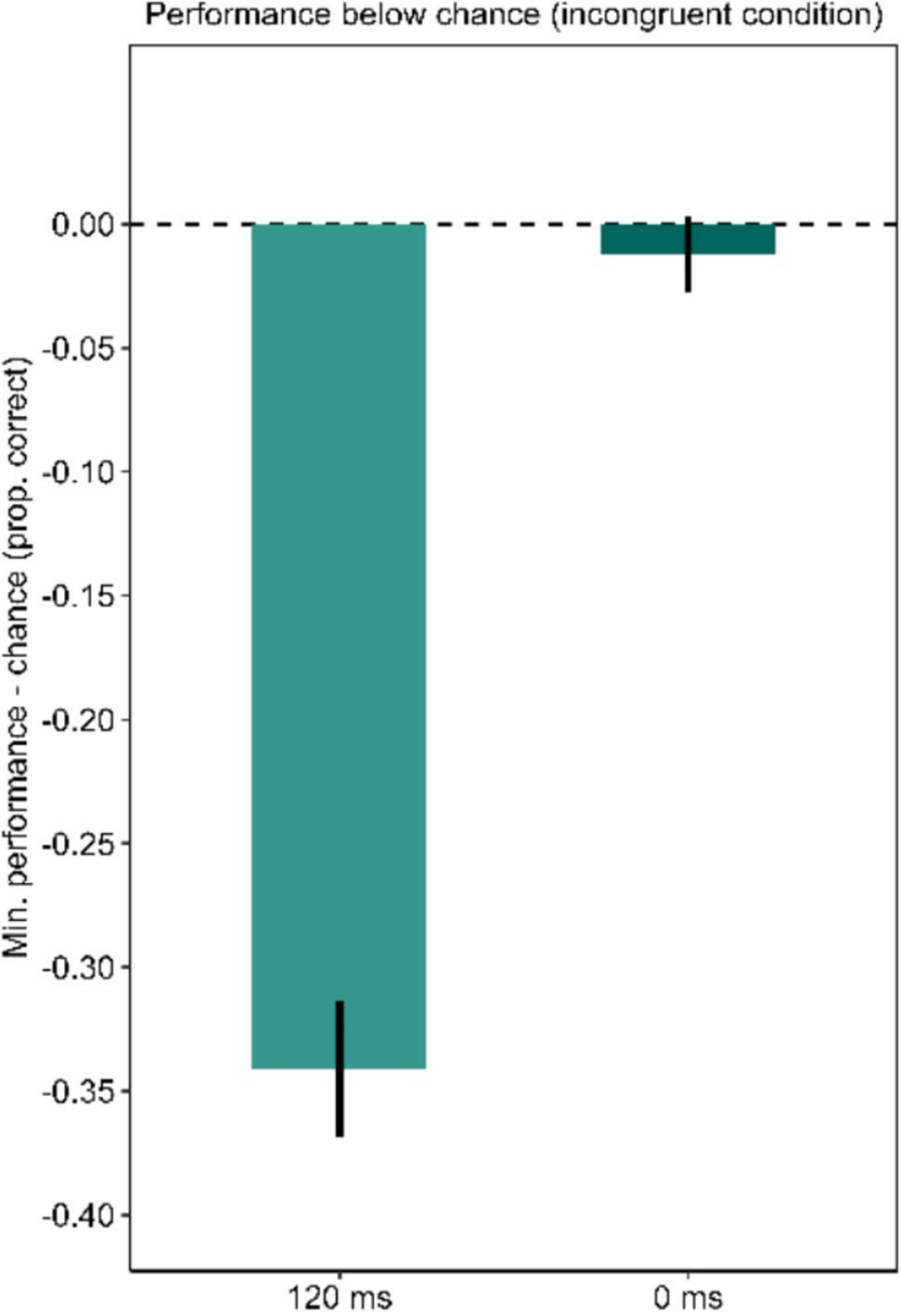
Urgency evoked a drop in performance below chance in the incongruent condition only in the 120-ms stimulus-onset asynchrony (SOA) condition. The figure shows the minimum of the performance below chance in the incongruent conditions for trials with 0-ms SOA and trials with 120-ms SOA. In the 120-ms SOA condition, performance dropped significantly deeper below chance. Error bars are standard errors from a bootstrap

Additionally, the minima of the incongruent functions of both SOA conditions were compared to check if the 120-ms SOA evoked a larger drop in performance below chance than the 0-ms SOA condition. The results are shown in Fig. 10. As expected, the performance in the 120-ms SOA condition dropped deeper below chance level to a minimum of 0.16 than the performance in the 0-ms SOA condition with a minimum of 0.49. The permutation test revealed a significant difference with p < 0.001.

These findings indicate that when a processing asymmetry between the distractors and the target is artificially generated, urgency heavily impairs goal-directed behavior. In a certain time-window, reactions are mainly driven by the identity of the irrelevant flanker stimuli instead of the target identity.

## Discussion

The present study aimed to investigate whether the effect of urgency on cognitive control can be transferred to cognitive control tasks that presumably evoke cognitive conflicts later, since they do not involve any processing asymmetry between the task-irrelevant information and the task-relevant information. In two Experiments, urgency was applied to two Eriksen flanker tasks, using color stimuli in Experiment 1 and letter stimuli in Experiment 2. In both experiments, urgency did not open up a time window in which the stimulus-driven information overpowered the goal-relevant information and thus dominated the response. In both experiments the tachometric function of the incongruent condition was similar to the function of the congruent condition. For both conditions, the performance fluctuated around chance level for negative as well as very short rPTs. With increasing rPT the performance also increased up to near-perfect performance. No significant difference in the minima of the curves was observed. The only difference that could be observed was a later increase in performance in the incongruent condition compared with the congruent condition in both experiments.

These results contradict previous studies regarding the effect of urgency on cognitive control (Krause & Poth, 2023; Poth, 2021; Salinas et al., 2019). Previous research revealed that urgency evoked a drop in performance below chance level in the incongruent condition. This result was concordant for an antisaccade task (Salinas et al., 2019), manual tasks like the Spatial Stroop task, the Simon task, and the Numerical Stroop task, and even a non-spatial Eriksen flanker task with arrow stimuli (Krause & Poth, 2023, 2025; Poth, 2021). Critically, all tasks that were previously used to investigate the effect of urgency on cognitive control were cognitive control tasks that led to early-onset cognitive conflicts, since they involved an asymmetry in processing speed between the conflicting stimulus information and the task-relevant information.

In an antisaccade task, as used by Salinas et al. (2019), a salient stimulus is presented on the right or on the left side of the screen (Hutton & Ettinger, 2006; Munoz & Everling, 2004). Participants are instructed to perform an eye movement in the opposite direction, away from the stimulus, although such a sudden onset of a stimulus automatically captures the gaze (Boot et al., 2005; Irwin et al., 2000; Theeuwes, 1991; Theeuwes et al., 1998). In the Spatial Stroop task, the conflicting dimension is the position of the stimulus (Lu & Proctor, 1995). An arrow stimulus is presented either on the left side or on the right side of the screen. Its position is irrelevant; participants are instructed to react to the pointing direction of the arrow. Here, a spatial cognitive conflict between the position and the meaning emerges (Lu & Proctor, 1995). In the Numerical Stroop task, the position is irrelevant for the task itself. Two numbers are presented, one positioned to the right and one to the left of the center of the screen (Dadon & Henik, 2017; Henik & Tzelgov, 1982). The participants should respond to the numerically larger number. The physical size of the number varied but should be ignored. Thus, a cognitive conflict between the numerical and the physical size of the numbers arises here (Henik & Tzelgov, 1982).

The delta plots of spatial cognitive control tasks, like the Spatial Stroop task or the Simon task, show a negative-going progress of the cognitive conflict (Pratte et al., 2010). This means, for fast RTs, the cognitive conflict is the highest. With increasing RT, the cognitive conflict decreases. This can be attributed to a processing asymmetry between the spatial position of the stimulus, which is a very salient feature and thus processed fast, and the relevant stimulus information. In a recent study, it could be shown that for visual perception the processing speed of spatial location is higher and processing starts earlier as compared with the object identity (Poth & Schneider, 2025). These findings point to a processing asymmetry and a natural preference of the visual system for processing space. Thus, in fast responses, the position is already processed, while the relevant dimension, i.e., the meaning or color of the stimulus, is processed later. With increasing RT, the processing of this relevant dimension also increases and thus the cognitive conflict is reduced. This may be transferred to the Numerical Stroop task in which the irrelevant information is the physical size, which is, like the position, very salient and processed faster than the stimulus, meaning the numerical size (Dadon & Henik, 2017; Henik & Tzelgov, 1982). Other cognitive control tasks, such as for example the color-word Stroop task, however, show a positive-going curve progression (Pratte, 2021). Here, the cognitive conflict is smaller for fast reactions. Fast responses are less influenced by the irrelevant stimulus dimension, whereas later responses are more influenced.

The only task in which the effect of urgency on cognitive control has been shown in a task that does not involve a natural processing asymmetry between the stimulus dimensions is the Eriksen flanker task from Experiment 2 in the study by Poth (2021). In an Eriksen flanker task the target stimulus is presented in the center of some other stimuli, the flanker stimuli, which can match the meaning of the target or deviate from it (Eriksen & Eriksen, 1974). In conflict situations, when the meanings of the stimuli differ, a cognitive conflict emerges between the goal-relevant reaction to the target and the task-irrelevant reaction to the flanker stimuli (Eriksen, 1995; Sanders & Lamers, 2002). Thus, this conflict is a conflict between the meanings of similarly processed stimuli.

Critically, in this study of Poth (2021), arrow stimuli were used. In multiple studies, it was shown that arrow stimuli automatically lead to a shift of attention in direction of their pointing direction and favor reactions that match this direction, even when the arrows are classified as task-irrelevant (Eimer, 1995; Hommel et al., 2001; Ristic & Kingstone, 2006). This reveals that arrows are overlearned stimuli that are highly associated with spatial directions. Regarding the arrow version of the Eriksen flanker task, it was shown that the delta plot, which illustrates the size of the congruency effect over the RT (De Jong et al., 1994), is an inverted U shape, which is more similar to spatial tasks, which normally show negative-leaning delta plots (Pratte, 2021; Pratte et al., 2010; R. Ridderinkhof, 2002). In Eriksen flanker tasks with, for example, color stimuli, an increasing function is observed (Pratte, 2021). This could explain why, in the present study, no dominance of the stimulus-driven response over the goal-driven response under urgency is elicited. Although the same task was used as by Poth (2021), the stimuli used differed. Since in an Eriksen flanker task with arrow stimuli there is also a large cognitive conflict for fast reactions, urgency here evokes a drop in performance below chance level in conflict situations. However, this is not the case for an Eriksen flanker task using stimuli that are processed on a higher level like colors or letters as used in the present study. In these tasks, there is no sufficiently large cognitive conflict for fast RTs to evoke a drop in performance below chance in urgent conflict situations, so that goal-directed behavior can be maintained.

To summarize, all tasks that were used so far to investigate the effect of urgency on cognitive control have in common that the stimulus-driven information used to drive the conflict has a fast and low-level processed stimulus property, such as position (antisaccade task, Spatial Stroop task, Simon task), a salient physical size (Numerical Stroop task), or an overlearned symbol with spatial associations (Eriksen flanker task with arrow stimuli). In addition, for all these tasks the conflicting stimulus dimension was closely related to the spatial response modality, favoring responses triggered by this irrelevant stimulus information rather than the task-relevant stimulus information. Thus, a (at least partially) negative delta plot can be assumed for all tasks, indicating that in all tasks an early-onset cognitive conflict was evoked. Thus, the effect of urgency on cognitive control seems to be driven by fast processed stimulus information and early cognitive conflicts.

This also corresponds with previous results, indicating that the size of the drop in performance below chance level evoked by urgency seems to depend on the processing speed of the irrelevant stimulus information. In their study, Salinas et al. (2019) manipulated the luminance and thus the salience of the target stimulus. The results showed that the size of the dip was larger for targets with higher luminance, indicating that the processing speed of the target plays a role in the size of the drop in performance below chance. Additionally, Krause and Poth (2023) showed that in a Spatial Stroop task a reduction in processing capacity that is directed towards the target influenced the size and form of the drop in performance below chance.

To test for the assumption that the urgency effect might depend on a processing asymmetry favoring the early processing of the conflicting visual stimuli, Experiment 3 was conducted, in which we artificially evoked a processing asymmetry between the flanker stimuli and the target stimulus by introducing a SOA between them. The results for the condition with an SOA of 0 ms, which corresponds to Experiments 1 and 2 in the methodology, replicated the results of these experiments. In the 120-ms SOA, however, the incongruent condition urgency evoked a dominance of the flanker-driven response over the task-driven response. Performance dropped clearly below chance level before it increased. These results confirmed the notion that the effect of urgency on cognitive control may be based on strong cognitive conflicts for short RTs due to a processing asymmetry with faster processing of the task-irrelevant information. This indicates that the effect of urgency on cognitive control may be based on a reinforcement of such processing asymmetries by urgency.

Because of the urgency manipulation, fast reactions are often required, so that in the decisive time window there are rather short RTs (Poth, 2021; Salinas et al., 2019). If a task or stimulus is used in which the irrelevant stimulus dimension is processed quickly, i.e., the curve in the delta plot is negative, this dimension influences the corresponding reaction and there is a strong cognitive conflict in high-urgency situations. In the case of irrelevant stimulus information that is processed later and for which the peak of the cognitive conflict is at later responses, i.e., the curve in the delta plot is positive, the responses in the relevant time period are not yet influenced by the irrelevant dimension, or are influenced to a lesser extent, and the cognitive conflict is lower. In such late cognitive conflicts that do not involve processing asymmetry, cognitive control can be maintained even under urgency. This can explain the absence of a drop in performance below chance level in Experiments 1 and 2 and the 0-ms SOA condition in Experiment 3.

As seen in the delayed rise of the incongruent tachometric function compared to the congruent curve in Experiments 1 and 2, a cognitive conflict also arises in the incongruent condition in these two experiments. This delayed rise of the incongruent curve reflects the classical congruency effect, which describes slower RTs in the incongruent condition. However, due to the equally processed stimuli, the conflict is not yet large enough to cause a dip under chance level in high-urgency situations, and cognitive control can be maintained. In later reactions, when the cognitive conflict may be stronger, the time pressure is no longer high enough, enough time remains for the response, and thus the correct reaction can be executed. Therefore, no dominance of the stimulus-driven response over the task-relevant response was evoked. Together with the results of Experiment 3, these results indicate that the effect of urgency on cognitive control is specific to tasks in which the stimulus-driven information is processed faster than the task-relevant information, so that an early cognitive conflict arises, such as is the case in spatial cognitive control tasks, or tasks using spatially associated stimuli.

Studies on delta plots have shown that the evolution of the cognitive conflict over the RT varies between tasks (Pratte et al., 2010). They have shown that cognitive conflicts are large for short RTs, particularly in tasks involving processing asymmetries (Burle et al., 2005; Pratte et al., 2010; Schwarz & Miller, 2012). While this study matches this results, it also reveals further insights into the effect of urgency on cognitive control. In comparison to classic cognitive control tasks, which usually find slower RTs in incongruent compared with congruent trials, studies on the effect of urgency on cognitive control do not only show that participants react slower or make more errors in the incongruent condition, but also that they perform below chance in urgent situations. This means that reactions are mainly dominated by the salient but irrelevant stimulus dimension in a certain time window, meaning participants mainly react contrary to their task-goal. The present study revealed that this effect is, however, not general to all cognitive control tasks, but is specific for tasks in which there is an asymmetry in processing speed with the irrelevant dimension of the stimulus being processed faster than the relevant dimension. This is, for example but not solely, the case for spatial cognitive control tasks, in which the spatial location of a stimulus conflicts its meaning. The present results indicate that urgency impacts performance by amplifying these processing asymmetries.

Due to the urgency approach, by analyzing the tachometric function, it is possible to investigate the contribution of the flanker stimuli and the target stimulus to the cognitive conflict over the time of the rPT with high temporal precision (cf. Salinas et al., 2019). Therefore, while the present study mainly indicates that due to the absent processing asymmetry in Eriksen flanker tasks, there is no clear processing advantage for the irrelevant flanker stimuli in urgent trials, it also provides further insights into the Eriksen flanker task itself. The tachometric functions show that for negative and short rPTs performance is around chance, meaning the reaction was not driven by a stimulus yet. In the time-window where the congruent tachometric function increases but performance in the incongruent function stays around chance, reactions seem to be driven by the flanker stimuli and the target equally. This differs from previous results that have shown that in a certain time-window reaction were dominated by the irrelevant stimulus feature (Krause & Poth, 2023; Poth, 2021; Salinas et al., 2019). Afterwards, with increasing rPT, the target is focused and preferably processed, so that the performance in the congruent condition also increases. This idea is in line with models that explain the flanker effect using the idea, that attention is at first captured by both the flanker stimuli and the target, but can be focused on the target over time (White et al., 2011). This is also closely related to the concept of biased competition (although it in general refers to ventral stream processing; Bundesen, 1990; Bundesen et al., 2005; Desimone & Duncan, 1995), which suggests that cortical receptive fields high up in the processing chain are wide in the beginning and thus attention is captured by all stimuli, but then shrink around and focus attention on the target. In addition, regarding conflict tasks in general, these results imply that differences in conflict task may be based on the strength of the processing asymmetry between the task-relevant and the task-irrelevant feature. In one of the most prominent theories regarding the classification of conflict tasks, Kornblum et al. (1990) suggested that the conflict task can be classified by the dimensional overlap between the stimulus features and the response dimension. However, the present results suggest that there are additional aspects that should be considered for such classifications, specifically the processing speed of the stimulus features and the presence of a processing asymmetry.

Taken together, the present findings reveal that urgency turns processing asymmetries into cognitive conflicts extremely vicious for human behavior. That is, while classic evidence showed that humans act more slowly in situations of cognitive conflict (Lu & Proctor, 1995; MacLeod, 1991), we observed that this changed under urgency: In urgent situations, a time-window is opened in which processing asymmetries led to cognitive conflicts taking over control of action and elicit behavior against the human will. As a result, behavioral errors are made, whose ultimate ramifications in everyday life could be fatal (e.g., in traffic situations).

## Conclusion

The present study showed that the effect of urgency on cognitive control occurs for conflicts including processing asymmetries and thus early arising cognitive conflict, leaving cognitive conflicts from equally fast processed stimulus-dimensions unaffected. As such, they suggest that urgency affects cognitive control by amplifying cognitive processing asymmetries.

## Supplementary Information

Below is the link to the electronic supplementary material.

Supplementary file1 (DOCX 328 KB)

## Data Availability

All data has been made publicly available on the Open Science Framework and can be accessed at https://osf.io/bgt36/

## Code availability

The experimental code and analysis code have been made publicly available on the Open Science Framework and can be accessed at https://osf.io/bgt36/

## Declarations

### Open Practices Statement

The data and materials for all experiments are available at the Open Science Framework (https://osf.io/bgt36/). All experiments were preregistered on the Open Science Framework (Experiment 1: https://osf.io/s4w3z; Experiment 2: https://osf.io/5qv2r; Experiment 3: https://osf.io/wydzq).
