# Supplementary material for: Urgency overpowers cognitive control by amplifying cognitive processing asymmetries
Source: Atten Percept Psychophys. 2025 Jul 13;87(6):1974–93. doi: 10.3758/s13414-025-03102-w (PMC12331796; doi:10.3758/s13414-025-03102-w)
Supplement: Supplementary file 1 — Supplementary file1 (DOCX 328 KB) [file 13414_2025_3102_MOESM1_ESM.docx]

**Supplemental Material**

We performed an additional analysis to investigate why performance fell below chance level in the congruent condition (i.e. the trials with an SOA of 0 ms) in Experiment 3. We computed separate tachometric functions for the two target stimuli that either required a left-hand or right-hand response. That is, participants were instructed to react to the target stimulus “S” with the left mouse button using their left index finger, and to the target stimulus “H”, with the right mouse button, using their right index finger. Since all participants are right-handed, we suspected there might be a preference for responding with the right mouse button. Such bias could influence performance especially for short rPTs, for which the target is visible too shortly for being used for response selection. The analyses of the separate tachometric functions show that the below-chance performance (for the SOA of 0 ms) indeed stemmed from trials with the target letter “S”, requiring a left-hand response. Performance in the incongruent condition is below chance for all rPTs until it rises toward a high performance level. In the congruent condition, there is a clear drop of performance below chance for short rPTs. In contrast, for the target letter “H” that required a right-hand response, performance was above chance throughout all rPTs instead (for the trials with an SOA of 0 ms). This could suggest a bias in favor of responding with the dominant right hand, explaining the below-chance performance in the congruent condition for trials with an SOA of 0 ms. However, it should be noted, that this is a post-hoc explanation. Further research is required to confirm this explanation.


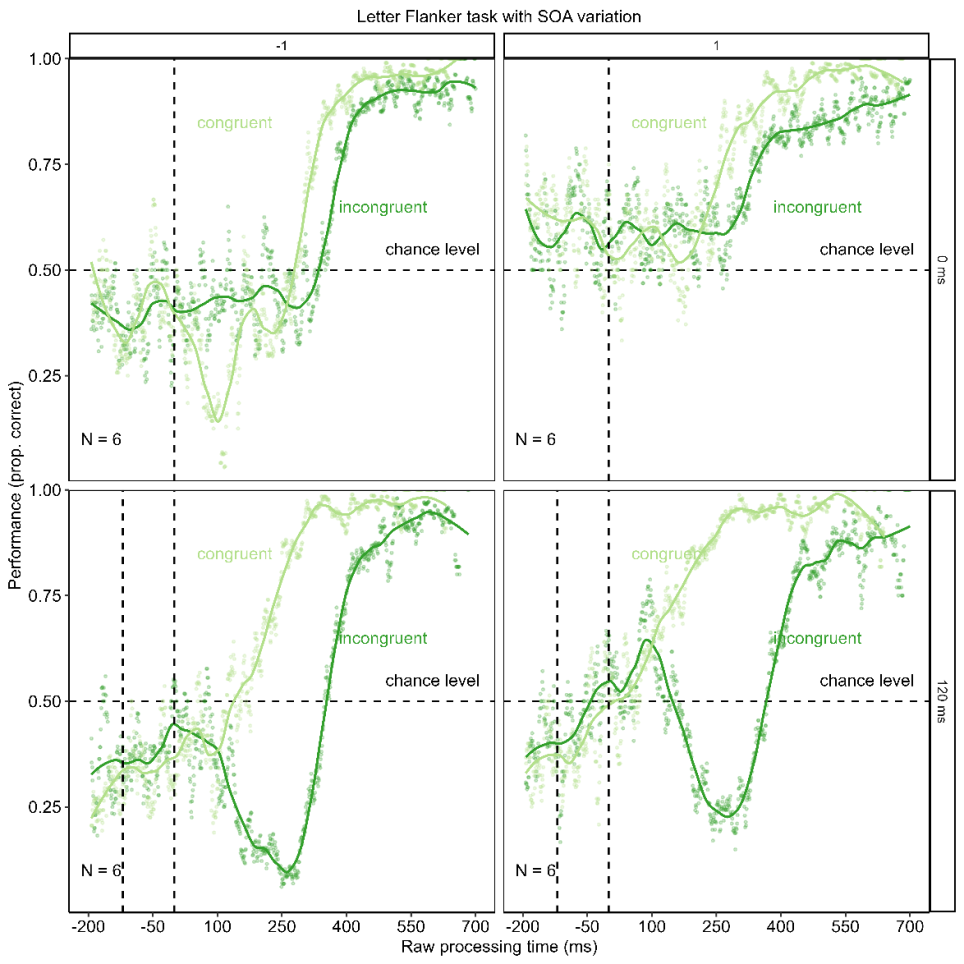


Figure S1. Tachometric functions of Experiment 3 separately for the different target stimuli.

This figure illustrates the tachometric functions of the congruent and the incongruent condition for both SOA conditions separately for target letter S (-1) and target letter H (1).
